# Cross-Cultural Differences in Gastrointestinal Symptoms and Sensory Responses in Individuals with Autism: A Comparison Between Spain and Colombia

**DOI:** 10.3390/children12070889

**Published:** 2025-07-06

**Authors:** Tíscar Rodríguez-Jiménez, Ignasi Navarro-Soria, Agustín E. Martínez-González

**Affiliations:** 1 Department of Psychology and Sociology, Faculty of Social and Human Sciences, University of Zaragoza, 44003 Teruel, Spain; 2 Department of Developmental Psychology and Didactics, University of Alicante, 03690 Alicante, Spain; ignasi.navarro@ua.es (I.N.-S.); agustin.emartinez@ua.es (A.E.M.-G.)

**Keywords:** autism, functional gastrointestinal disorders, gastrointestinal symptoms, constipation, pain, cross-cultural

## Abstract

Background: Between 40 and 70% of the population with autism have been found to suffer from functional gastrointestinal disorders (FGIDs). The emergence of FGIDs is related to lower quality of life and greater medical resources, somatization and emotional instability. There is a paucity of research available that examines gastrointestinal symptoms and sensory responses in individuals with autism in different countries and cultures. The aim of the present study is to compare the possible differences between gastrointestinal symptoms and sensory reactivity between two samples of individuals with autism from Spain and Colombia. Methods. Differences in gastrointestinal symptoms and sensory response were analysed between individuals with autism from Spain (*n* = 65; mean age = 8.91, SD = 4.02) and Colombia (*n* = 62; mean age = 10.16, SD = 5.31). Results. No differences were found as a function of age, sex and autism severity between Spanish and Colombian participants. More severe functional nausea and vomiting was reported by the Colombian sample when compared with the Spanish sample (*p* < 0.00, *d* = 0.42). Similarly, greater sensory reactivity emerged in Colombian individuals with autism relative to Spanish individuals with autism. Conclusions. Due to methodological limitations, sample size and other factors that could not be analysed in this research, it is not possible to draw conclusions about the influences of cultural or biological factors on gastrointestinal symptomatology and sensory reactivity among both autistic populations. This work could encourage more rigorous cross-cultural research in the future.

## 1. Introduction

Autism spectrum disorder (ASD) is a neurodevelopmental disorder that is mainly characterised by impaired communication and repetitive behaviour [1]. In addition, ASD diagnosis is associated with a number of comorbid neurodevelopmental disorders such as intellectual disability (ID) [1].

The 2016 Rome IV criteria advocate for considering functional gastrointestinal disorders (FGIDs) as a set of chronic or recurrent gastrointestinal symptoms (GS) which have a gut–brain interaction [2]. Further, FGIDs were associated with lower quality of life and more frequent visits to the doctor [3], higher medical expenses, increasing somatization [4] and psychosocial difficulties [5].

Gastrointestinal symptom (GS) incidences of between 40 and 70% have been found in autistic populations [6,7]. A recent meta-analysis reported that 37% of children with ASD suffered from constipation, whilst 21% suffered from abdominal pain, 19% from diarrhoea, 8% from vomiting and 23% from abdominal distension [8]. Selective or restrictive dietary patterns (e.g., picky eaters) are related to sensory response and GS in individuals with ASD [6,9]. Thus, GS such as constipation are more severe in individuals with ASD than neurotypical controls [6]. Further, significantly higher rates of children with ASD and GS report both anxiety and sensory over-responsivity (SOR) [10]. Similarly, associations have been found between anxiety, SOR and chronic abdominal pain, with SOR being a significant predictor of the onset of pain [11]. Thus, abdominal pain appears to be common amongst children with ASD. These results indicate that anxiety, SOR, GS and abdominal pain are possibly interrelated and may have common underlying mechanisms [10,11]. Several studies have highlighted the existence of a relationship between abdominal pain, selective dietary patterns, emotional instability and gut dysbiosis in neurodevelopmental disorders such as ASD. Notably, all of these aforementioned variables seem to be related to the gut–microbiota–brain axis through the enteric nervous system [6,7,12,13,14].

There is a lack of cross-cultural research available examining differences in ASD symptoms between countries [15]. Some of these meta-analyses include original studies with samples of fifty individuals with ASD, and this aspect is more evident in countries that are initiating their first research (e.g., India, Turkey, etc.) [6,7]. In fact, so far, no data have been shown for Colombia, and this is an aspect to highlight given the scarcity of studies in Latin America. Furthermore, this lack of research becomes more evident when differences in GS, a variable closely related to diet, and epigenetic factors are considered [8]. Cross-cultural differences in GS and sensory reactivity between countries have not been analysed, with this shortfall being even more stark in Latin and Hispanic countries. The aim of the present study, therefore, was to examine differences in gastrointestinal symptoms and sensory response between individuals with autism from Spain and Colombia.

## 2. Materials and Methods

### 2.1. Participants

A sample of 127 (65 Spanish and 62 Colombian) individuals with ASD and their families participated in the survey. Participants came from all regions of Spain and Colombia. Participating children were enrolled at centres delivering special education, early childcare or day care, or regular schools with open classrooms. Spanish participants came from different-sized urban and rural areas in the regions of Valencia, Murcia and Andalusia. The Colombian sample came from the Andean, Caribbean, Orinoquía and Pacific regions.

DSM-5 diagnostic criteria for ASD [1] served as the referent for inclusion of individuals in both the Spanish and Colombian samples. Spanish participants had previously been diagnosed by mental health services and pertinent institutions responsible for ascertaining the degree of disability and dependency. Similarly, autism diagnoses in Colombian participants were made by public and private mental health institutions (e.g., early intervention centres, autism centres, etc.).

Individuals with other concomitant diagnoses, such as motor disabilities, multiple disabilities, attention-deficit/hyperactivity disorders, obsessive-compulsive disorders, neurodegenerative diseases and mental illnesses, were excluded. Understandably, participants with ASD and ID were included when ASD was the primary diagnosis.

### 2.2. Measures

#### 2.2.1. Sociodemographic Questionnaire

Lam and Aman’s [16] sociodemographic questionnaire was adapted for the present online study. This tool consists of a series of questions about sociodemographic characteristics (e.g., age, sex, country of birth) and comorbidities (e.g., intellectual disability).

#### 2.2.2. Clinical Questionnaire on Gastrointestinal Symptoms

This is an ad hoc questionnaire that was developed to identify gastrointestinal disorders according to Rome criteria [2]. It consists of a series of questions about gastrointestinal disorders (e.g., diarrhoea, abdominal pain, dyspepsia and gastroesophageal reflux).

#### 2.2.3. Gastrointestinal Symptom Severity Scale (GSSS)

This instrument is based on Rome IV criteria [2] and consists of seven items pertaining to main gastrointestinal symptoms (constipation, diarrhoea, average stool consistency, stool odour, flatulence and gas and abdominal pain). The instrument comprises an abdominal subscale (abdominal pain, gas and constipation) and a vomiting and defecation subscale (vomiting, defecation in inappropriate places, diarrhoea, rumination). Items are rated along a four-point Likert scale ranging from 0 (none/nothing or this symptom does not occur) to 3 (very frequent and troublesome). The GSSS presents adequate psychometric properties in individuals with autism and in neurotypical children and adolescents [17,18,19]. Internal consistency coefficients of 0.73 have been reported in children with typical development [19], whilst coefficients between 0.75 and 0.61 have been reported in individuals with autism [18]. Two versions of the instrument are available, namely, a version for caregivers-professionals and a self-report version. The caregivers-professionals version of the test was administered in the present study. Internal consistency for the overall GSSS were 0.64 in the Spanish sample and 0.60 in the Colombian sample.

#### 2.2.4. Pain and Sensitivity Reactivity Scale (PSRS)

The PSRS is a tool that evaluates reactivity to pain and sensory reactivity according to 50 items. It is composed of three dimensions, namely, pain, sensory hyporeactivity and sensory hyper-reactivity. Items are rated on a four-point Likert scale ranging from 0 (behaviour does not occur) to 3 (behaviour occurs and is a severe problem). Both hyposensitivity and hypersensitivity dimensions comprise tactile, olfactory, visual, gustatory and auditory items. In addition, the PSRS includes a pain reactivity domain which comprises seven items. The PSRS was elaborated based on theoretical requisites conceived by Miller et al. [20], which characterises sensory modulation disorders according to three patterns (hyper-response, hypo-response and sensory seeking) as proposed nosology for diagnosis. Two versions of the PSRS are available. The first is a version administered for completion by caregivers and professionals, whilst the second is a self-report version [21]. The caregiver version of the PSRS has shown excellent internal consistency in samples with ASD (pain α = 0.83; broad sensory hyporeactivity α = 0.90; broad sensory hyperreactivity α = 0.93) [22]. The caregiver version was used in the present study. Internal consistency values for the Spanish sample were 0.57 for pain, 0.84 for broad sensory hypo-reactivity, 0.93 broad sensory hyper-reactivity and 0.93 for overall PSRS. On the other hand, internal consistency values for the Colombian sample were 0.80 for pain, 0.87 for broad sensory hypo-reactivity, 0.94 broad sensory hyper-reactivity and 0.95 for overall PSRS.

### 2.3. Procedures

The present study was approved by the Ethics Committee of the University of Alicante in Spain (reference: UA-2019-10-04) and the Catholic University of Pereira in Colombia (reference: UCP-2021-05). The survey was carried out using online data collected from the parents and caregivers of individuals with ASD who provided written informed consent. The reporting assessment protocol was individually applied through online survey tools, specifically, LimeSurvey (LimeSurvey GmbH, Hamburg, Germany), in Spain, and Formsite (Downers Grove, IL, USA), in Colombia. Both platforms complied with the same comprehensive autism assessment protocol.

For the recruitment of all participants, a cover letter containing study information was sent to ordinary centres, special education centres, associations serving families with children with autism, etc. Centre management teams informed families about the study and provided access to the online survey. Centres held an online or telematic meeting in order to timetable appointments and explain the purpose of the research. Subsequently, participating institutions contacted families to organise a meeting and further explain the purpose of the study. Similarly, some institutions provided the research team with contact information so that study researchers could directly explain the purpose of the study to families. Finally, an explanatory video was shared using social networks. All participating families and caregivers had a child diagnosed with ASD according to DSM-5 criteria [1]. Individuals with ASD, whether with or without ID, were diagnosed according to DSM-5 criteria using standardised scales (e.g., Wechsler nonverbal scale of ability, Leiter-3 scale, etc.). Participants had all been previously diagnosed by the pertinent mental health services and institutions responsible, in their given country, for establishing their degree of disability and dependency. Families with children with another type of neurodevelopmental disorder (e.g., ADHD) or with a diagnosis of ASD but who had not been assessed for possible ID were excluded from the study.

With regard to individuals with ASD in Spain, diagnoses were made at early care centres and through regional paediatric services attached to local mental health centres [23]. Common standardised scales were used to reach a diagnosis of autism (e.g., communication domain of the Vineland adaptive behaviour scales, Vineland adaptive behaviour scales, autism diagnostic interview-revised, autism diagnostic observation schedule–generic, childhood autism rating scale, etc.). However, the diagnostic process for individuals with ASD in Colombia was applied in line with the “clinical protocol for the diagnosis, treatment and comprehensive care route for boys and girls with autism spectrum disorders” outlined by the Colombian Ministry of Health [24]. This national protocol uses applied behaviour analysis (ABA) to reach clinical and non-psychometric diagnoses. A limitation of this protocol is that most of the standardised scales used to apply it have not yet been validated, and scales taken adapted from measures used with Spanish or American populations are typically used. Study researchers organised a training session for all participating centres, in which they describe in detail the purpose of the research study, the tests to be used and the process to be followed during test administration.

Expert psychologists and similar professionals (educational psychologists, special education teachers, psychologists) were engaged in the administration of online tests to participating families, which sought to gather information on the perceptions and knowledge of individuals with ASD at the participating institutions that served them. Psychologists based at the participating centre were on hand to help families resolve any doubts regarding their diagnosis in the first part of the survey. The explanatory video mentioned above urged families to consult pertinent psychological and psychiatric reports should any doubts exist regarding their diagnosis.

The linguistic adaptation process adhered to international guidelines. Initially, the translator of two Colombian linguistic experts translated the instrument from Spanish into Colombian Spanish and assessed the difficulty of the translation, indicating suggestions for changes in some items. The changes needed for each item were based on the following: (1) no changes were needed; (2) modifications were needed to maintain semantic and conceptual equivalence. Subsequently, a back-translation from Colombian Spanish to Spanish was carried out by a Colombian professor who was living in Spain for his doctoral thesis and was unaware of the original Spanish version. The European Spanish versions of the GSSS and PSRS were revised by three specialist Colombian psychologists and one Spanish psychologist who corroborated to ensure that items were culturally equivalent. This team reached consensus on an initial Spanish version of the scale. Subsequently, clarity and ease of understanding of all items were verified in a pilot study conducted with ten participants in Pereira (Colombia). To ensure comprehension, cognitive interviews were conducted with ten parents. The final version of the Colombian version of the instrument required no additional modifications after cognitive interviews with 10 primary caregivers. This revealed that no issues emerged regarding understanding, and so no linguistic adaptation was needed.

### 2.4. Data Analyses

Statistical analyses were performed using IBM SPSS Statistics v 29.0 for Windows [25]. Means and standard deviations for all items corresponding to all tests were calculated for both samples from the direct scores reported by participants. Cronbach’s alpha scores were calculated for all individual subscales and overall scores pertaining to the GSSS and PSRS, as a function of country. Next, as a preliminary step prior to performing the main analysis, sociodemographic variables were compared between the two countries in order to examine potential confounding factors. Chi-square analyses were conducted of the categorical variables of gender, age, comorbidity with intellectual disability (ID) and gastrointestinal symptoms. For the comparison of mean values and correlations, the Kolmogorov–Smirnov test was applied to verify the normality assumption for the use of parametric tests within each group. Outcomes revealed that the normality assumption was not satisfied. As a result, the non-parametric Mann–Whitney U test was used to determine whether GSSS and PSRS subscale and overall scores differed between Spanish and Colombian participants, with significance being set at *p* < 0.05. Effect sizes associated with these differences were calculated to determine whether statistically significant differences existed between the proportions reported in each country. In this sense, 0.20 ≤ *d* ≤ 0.50 represented a small effect size, whilst 0.51 ≤ *d* ≤ 0.79 corresponded to a medium effect size and *d* ≥ 0.80 reflected a large effect size [26]. Further, non-parametric Spearman’s rho coefficients were calculated.

To account for multiple comparisons, the Benjamini–Hochberg procedure (FDR) was used to adjust *p*-values throughout the analyses, thereby controlling the false discovery rate and preserving statistical power.

## 3. Results

Sociodemographic data pertaining to all 127 participants with ASD from Spain (*n* = 65) and Colombia (*n* = 62) are presented in Table 1. No differences were found in relation to sex (χ^2^ = 0.00; *df* = 1; *p* = 0.96) and age when comparing Spanish and Colombian samples with ASD (χ^2^ = 0.91; *df* = 1; *p* = 0.34). Outcomes also indicated largely similar outcomes regarding the frequency of ASD diagnosis without ID and with mild ID (χ^2^ = 1.58; *df* = 3; *p* = 0.66).

### 3.1. Differences in Gastrointestinal Symptoms

A higher rate of gastrointestinal disorders was found in the Colombian sample of individuals with ASD relative to the Spanish sample. Specifically, infectious diarrhoea and gastroesophageal reflux was three times higher in the Colombian sample. However, a higher percentage of abdominal pain was seen in the Spanish sample of individuals with ASD (see Table 2).

Outcomes regarding differences in gastrointestinal symptom severity between individuals with ASD from Spain and Colombia are presented in Table 3. With regard to gastrointestinal symptoms, the Colombian sample reported significantly higher mean scores on the functional nausea and vomiting subscale of the GSSS, with the magnitude of this difference being moderate.

The mean of both samples exceeds the cut-off point of the total GSSS, which is 2 points. Thus, both samples have a mean level of severity of gastrointestinal symptoms [18].

To account for multiple comparisons, the Benjamini–Hochberg procedure (FDR) was applied to the *p*-values. After adjustment, only the difference in the functional nausea and vomiting subscale remained statistically significant.

### 3.2. Differences in Sensory Response

Outcomes reveal that differences do not exist in the degree of pain experienced by Colombian and Spanish individuals with ASD. However, significantly higher scores were provided by Colombian participants when compared with Spanish participants with regard to sensory hyporeactivity and sensory hyper-reactivity PSRS subscale scores and overall PSRS scores. Furthermore, differences were found in all dimensions of sensory hyper-reactivity (tactile, olfactory, visual, taste and auditory). The magnitude of these differences was found to be moderate to high (see Table 4).

At the clinical level, both samples obtained a medium-low level of pain sensation according to the PSRS for the ASD population. Therefore, no major difficulties in pain sensation were observed. Likewise, both samples have obtained a mild level of sensory hyporeactivity at the clinical level. However, sensory hyperactivity has moderate levels at the clinical level in the Colombian sample compared to mild levels in the Spanish sample, following the PSRS [22].

To control for multiple comparisons, the Benjamini–Hochberg procedure (FDR) was applied to the *p*-values. After adjustment, significant differences remained for Total Hypo, Hypo-Visual, Hypo-Auditory, Total Hyper, Hyper-Tactile, Hyper-Olfactory, Hyper-Visual, Hyper-Taste, Hyper-Auditory and Total PSRS, while the remaining comparisons did not reach statistical significance.

### 3.3. Relationships Between Gastrointestinal Symptoms and Sensory Reactivity

In both the Spanish and Colombian samples, moderate associations were found between gastrointestinal symptoms and sensory reactivity (see Table 5).

A moderate correlation has been found between gastrointestinal symptoms, sensory hypo-reactivity and sensory hyper-reactivity in Spanish ASD individuals. However, the correlation of these variables was low in the Colombian sample.

## 4. Discussion

Descriptive outcomes reported in the present study indicate that the prevalence of gastrointestinal disorders in autistic individuals from Spain and Colombia was around 50%. This finding mirrors that found in previous studies [6,7,8]. Similarly, the proportion of participants in the Spanish sample suffering from abdominal pain was similar to that previously reported in the existing literature [8], with rates of diarrhoea in the Colombian sample also being similar to those reported by a previous study [8].

A higher prevalence of disorders related to the expulsion of food, namely, nausea, vomiting, diarrhoea and reflux was found in the Colombian sample with ASD recruited in the present study. Consistent with this finding, more severe levels of functional nausea and vomiting have been found in Colombian individuals with ASD compared to Spanish individuals with ASD in the GSS. However, differences were moderate in the nausea subscale of the GSSS and no statistically significant differences were observed in the total GSSS and in the abdominal pain subscale. Therefore, possible differences in gastrointestinal symptoms between the two countries should be taken with caution. Possible GS differences between the two countries may be due to multiple factors that have not been analysed in the present study: different food culture, meal times, ways of cooking, the composition of gut microbiota, etc. [27]. This possibility must be considered in light of the fact that the present study included both urban and rural populations from Colombia. This is important because previous studies found dysbiosis in the intestinal microbiome of an indigenous population with frequent episodes of gastrointestinal infections [28]. On the other hand, moderate but questionable internal consistency was found for the GSSS items in both samples. These results could be due to the small sample size, but this is an important limitation in this study.

In addition, present findings reveal statistically significant differences in sensory reactivity between the Colombian population with ASD and the Spanish population, being at more severe in the Colombian ASD population a clinical level, and with a high magnitude of differences between both samples. This finding is in line with that reported by a previous study, which found higher levels of repetitive behaviour in Colombian individuals with ASD [15]. Repetitive behaviour and sensory reactivity are highly interconnected with regard to ASD [1]. The higher level of sensory reactivity seen in Colombian individuals in the present study may be due to various aspects, such as the resources available for sensory stimulation at Colombian centres and treatment approaches [15], which could not be considered in the present study.

The results of this study support the relationship between gastrointestinal symptoms, pain and sensory reactivity [10,11]. However, correlations were moderate to low in the Spanish ASD individuals, and low in the Colombian sample. Correlation patterns are stronger between gastrointestinal symptoms and sensory reactivity in Spanish versus Colombian samples. This may raise a new hypothesis about different underlying mechanisms of these variables between countries, or factors related to the measurement properties of the instruments included in this study.

Ultimately, findings indicate that Colombian children with autism have greater gastrointestinal difficulties and greater sensory hyper-reactivity. It is often surmised that such symptoms are associated with alterations of the gut-microbiota [14]. However, present findings are of limited scope. The present study has a number of limitations that should be taken into consideration: (1) Although cross-cultural and cross-diagnostic comparative studies with autism have used similar samples [15], the size of both samples is small to be able to generalise the results. (2) The assessment methodologies to determine the diagnosis of ASD between Spain and Colombia are different. In this sense, in Colombia there is a scarcity of validation studies of psychometric instruments to determine the diagnosis of ASD [15]. Therefore, although an attempt has been made to homogenise both samples, there may be an important bias because there may not be comparable diagnostic entities between the two samples. Similarly, GSSS and PSRS instruments have not yet been validated in Colombian population with and without autism. On the other hand, the low internal consistency of the GSSS for both populations indicates that there are measurement issues that small samples cannot adequately address. Methodological limitations of this study include that there may be different cultural conceptualisations of symptoms, and different thresholds for reporting problems. In addition, the use of different online platforms (LimeSurvey vs. Formsite) introduces another potential bias (e.g., interface design, question presentation, etc.). (3) A larger sample size would have been desirable for the pilot test of the language adaptation of the instruments to Colombian Spanish. (4) Due to the sample size, it was not possible to perform measurement invariance analyses. (5) Variables related to the type of food or diet in the families of both countries have not been taken into account. (6) Different online platforms used could introduce measurement bias. (7) The gut microbiota of the individuals with ASD has not been analysed, so it is not possible to establish a direct relationship between these results and a possible dysbiosis or alteration of the gut microbiota in the sample of Colombian individuals with ASD. (8) Finally, this study does not take into account variables that may moderate the results found, such as health systems, socio-economic factors, educational resources, environmental exposures, dietary patterns and cultural attitudes towards disability across countries.

Therefore, it is urged that future studies include the following: (1) an analysis of the pyrosequencing of the 16S rRNA and comparison of samples taken from different countries; (2) a larger sample, although the recruited sample was similar in size to those used by previous studies [8]; and (3) additional variables such as the type of diet followed or food consumed.

In conclusion, this work could encourage more rigorous cross-cultural research on gastrointestinal symptoms and sensory reactivity in autism, using validated instruments and larger and more representative samples. Therefore, it is not possible to guarantee that the differences found are due to cultural or biological factors. The findings are preliminary evidence that justifies further research in this area of research.

## Figures and Tables

**Table 1:** Sociodemographic and diagnostic characteristics of the sample.

	Spanish Sample		Colombian Sample	
*N*	65		62	
Age (M/SD)	8.91 (4.02)		10.16 (5.31)	
Sex (male/female)	(48; 73.8%/ 17; 26.2%)		(46; 74.2%/ 16; 25.8%)	
Reported diagnosis	N	%	N	%
ASD w/o ID	50	76.9	45	72.6
ASD w Mild ID	9	13.8	10	16.1
ASD w Moderate ID	5	7.7	7	11.3
ASD w Severe ID	1	1.5	0	0
Context	N	%	N	%
Regular class in a regular school	43	66.2	42	67.7
Special class in a regular school	15	23.1	9	14.5
Special School	2	3.1	1	1.6
Other (e.g., residence, day centre, etc.)	5	7.7	10	16.2

Note. w = with; w/o = without; ASD = autism spectrum disorder; ID = intellectual disability; M = mean; SD = standard deviation.

**Table 2:** Presence of gastrointestinal disorders in the individuals with ASD according to the country.

Gastrointestinal Disorders	Spanish Sample (%)	Colombian Sample (%)	χ^2^	*df*	*p*
Diagnosis of gastrointestinal disease	52.3	59.7	0.70	1	0.40
Infectious diarrhoea	4.6	16.1	4.58	1	0.03 *
Nonspecific abdominal pain	20	9.7	2.66	1	0.10
Dyspepsia	9.2	4.8	0.93	1	0.34
Gastroesophageal reflux	4.6	21	7.71	1	0.01 *
Significant flatulence	9.2	6.5	0.34	1	0.56
Irritable bowel syndrome	3.1	0	1.94	1	0.16
Dyschezia	0	0	-	-	-
Inflammatory bowel disease	0	3.2	2.13	1	0.14
Celiac disease	0	0	-	-	-
Ulcerative colitis	1.5	0	0.96	1	0.33
Peptic ulcer disease	4.6	1.6	0.94	1	0.33
Crohn’s disease	0	0	-	-	-

Note. * *p* < 0.05.

**Table 3:** Differences in gastrointestinal symptom severity of individuals with ASD according to the country.

GSSS	Spanish Sample	Colombian Sample				
	*M* (*SD*)	*M* (*SD*)	*U*	*p*	*d* [95% CI]	FDR
Abdominal pain and defecation subscale	1.78 (2.13)	1.92 (1.88)	1829.50	0.35	0.07 [−0.28, 0.42]	0.40
Functional nausea and vomiting subscale	0.45 (0.93)	0.94 (1.34)	1499.00	0.00 **	0.42 [0.07, 0.78]	0.00 **
Total GSSS	2.23 (2.53)	2.85 (2.67)	1694.50	0.11	0.24 [−0.11, 0.59]	0.13

Note. GSSS = gastrointestinal symptom severity scale; M = mean; SD = standard deviation; ** *p* < 0.01.

**Table 4:** Differences in pain and sensory response of individuals with ASD according to the country.

PSRS	Spanish Sample	Colombian Sample				
	*M* (*SD*)	*M* (*SD*)	*U*	*p*	*d* [95% CI]	FDR
Pain	5.83 (2.73)	6.56 (3.66)	1895.00	0.55	0.23 [−0.12, 0.57]	0.55
Total Hypo	14.49 (9.59)	19.29 (10.74)	1438.50	0.00 **	0.47 [0.12, 0.82]	0.00 **
Hypo-Tactile	4.06 (2.98)	5.19 (3.61)	1663.00	0.08	0.34 [−0.01, 0.69]	0.10
Hypo-Olfactory	2.46 (2.73)	2.48 (2.42)	1894.00	0.55	0.01 [−0.34, 0.36]	0.55
Hypo-Visual	2.68 (2.43)	3.98 (2.70)	1379.00	0.00 **	0.50 [0.15, 0.86]	0.00 **
Hypo-Taste	2.38 (2.49)	3.13 (2.70)	1652.50	0.07	0.29 [−0.06, 0.64]	0.10
Hypo-Auditory	2.91 (2.28)	4.50 (2.64)	1312.00	0.00 **	0.64 [0.28, 1]	0.00 **
Total Hyper	16.66 (12.48)	26.40 (14.99)	1245.00	0.00 **	0.70 [0.34, 1.06]	0.00 **
Hyper-Tactile	4.22 (3.41)	6.52 (4.12)	1327.00	0.00 **	0.61 [0.25, 0.96]	0.00 **
Hyper-Olfactory	2.49 (2.87)	4.11 (3.64)	1499.00	0.01	0.49 [0.14, 0.84]	0.02 *
Hyper-Visual	1.86 (2.69)	3.37 (2.77)	1240.00	0.00 **	0.55 [0.19, 0.90]	0.00 **
Hyper-Taste	3.91 (3.26)	5.53 (3.61)	1477.50	0.00 **	0.47 [0.12, 0.82]	0.00 **
Hyper-Auditory	4.18 (3.39	6.87 (3.67)	1199.50	0.00 **	0.76 [0.40, 1.12]	0.00 **
Total PSRS	36.98 (20.79)	52.26 (25.56)	1315.00	0.00 **	0.65 [0.30, 1.01]	0.00 **

Note. PSRS = pain and sensitivity reactivity scale; Total Hypo = total sensory hyporeactivity; Total Hyper = total sensory hyperreactivity; M = mean; SD = standard deviation; * *p* < 0.05; ** *p* < 0.01.

**Table 5:** Correlations between gastrointestinal symptoms and sensory reactivity.

	PSRS Pain	PSRS Hypo	PSRS Hyper
Spanish sample			
GSSS Total	0.38 **	0.48 **	0.44 **
Colombian sample			
GSSS Total	0.33 **	0.33 **	0.27 *

Note. GSSS = gastrointestinal symptom severity scale; PSRS = pain and sensitivity reactivity scale; Hypo = sensory hyporeactivity; Hyper = sensory hyperreactivity; * *p* < 0.05; ** *p* < 0.01.

## Data Availability

The original contributions presented in this study are included in the article. Further inquiries can be directed to the corresponding author.
